# Bat Response to Differing Fire Severity in Mixed-Conifer Forest California, USA

**DOI:** 10.1371/journal.pone.0057884

**Published:** 2013-03-06

**Authors:** Michael R. Buchalski, Joseph B. Fontaine, Paul A. Heady, John P. Hayes, Winifred F. Frick

**Affiliations:** 1 Department of Biological Sciences, Western Michigan University, Kalamazoo, Michigan, United States of America; 2 School of Environmental Science, Murdoch University, Perth, Western Australia, Australia; 3 Central Coast Bat Research Group, Aptos, California, United States of America; 4 Department of Wildlife Ecology and Conservation, University of Florida, Gainesville, Florida, United States of America; 5 Department of Ecology and Evolutionary Biology, University of California Santa Cruz, Santa Cruz, California, United States of America; University of Western Ontario, Canada

## Abstract

Wildlife response to natural disturbances such as fire is of conservation concern to managers, policy makers, and scientists, yet information is scant beyond a few well-studied groups (e.g., birds, small mammals). We examined the effects of wildfire severity on bats, a taxon of high conservation concern, at both the stand (<1 ha) and landscape scale in response to the 2002 McNally fire in the Sierra Nevada region of California, USA. One year after fire, we conducted surveys of echolocation activity at 14 survey locations, stratified in riparian and upland habitat, in mixed-conifer forest habitats spanning three levels of burn severity: unburned, moderate, and high. Bat activity in burned areas was either equivalent or higher than in unburned stands for all six phonic groups measured, with four groups having significantly greater activity in at least one burn severity level. Evidence of differentiation between fire severities was observed with some *Myotis* species having higher levels of activity in stands of high-severity burn. Larger-bodied bats, typically adapted to more open habitat, showed no response to fire. We found differential use of riparian and upland habitats among the phonic groups, yet no interaction of habitat type by fire severity was found. Extent of high-severity fire damage in the landscape had no effect on activity of bats in unburned sites suggesting no landscape effect of fire on foraging site selection and emphasizing stand-scale conditions driving bat activity. Results from this fire in mixed-conifer forests of California suggest that bats are resilient to landscape-scale fire and that some species are preferentially selecting burned areas for foraging, perhaps facilitated by reduced clutter and increased post-fire availability of prey and roosts.

## Introduction

Disturbance-habitat dynamics are widely understood to play central roles in the conservation of animal populations. For example, the provision of heterogeneous late-successional habitat for species of conservation concern like fisher (*Martes pennanti*) and spotted owl (*Strix occidentalis*) in western North America is mediated by disturbance history, primarily fire and human management [1], [2], [3]. Fire extent, intensity, and frequency in forests shapes the spatial distribution of successional stages [4], plant species composition [5], and availability of standing and downed wood [6]; all of which influence the abundance and distribution of wildlife [7], [8], [9]. Current understanding of wildlife response to fire events in North America is based almost entirely upon studies of a limited number of bird and small mammal species, limiting the ability of forest managers to anticipate wildlife population dynamics following wildfire, or in relation to fire severity [9].

Large forest fires create heterogeneous post-fire landscapes [10] suggesting that mixed-severity fire may be the norm rather than the exception [11], [12]. In step with this emerging paradigm, researchers have begun to investigate the response of wildlife to mosaics of burn damage, with evidence of the importance of wildfire-maintained habitats [8] and resilience of late successional-associated species to mixed-severity fire (e.g., California Spotted Owl, [1]). The notion of fire mosaics supporting greater faunal diversity has also been advanced though evidence supporting this hypothesis is lacking or contradictory, particularly for vagile species able to move across habitat edges [13], [14].

Bats are a major component of wildlife communities in forest ecosystems, representing approximately one quarter of global mammalian diversity [15]. Yet little is known regarding the effects of wildfire on bat species [9], [16], [17]. Existing knowledge of bat response to forest disturbance is largely from studies of ecological thinning [18], various levels of harvest [19], [20], [21], [22], [23], or prescribed burning [24], [25], [26]. Such studies have shown that activity of bats increases following disturbance, with increased activity attributed to three possible causes. First, disturbance potentially increases foraging habitat quality by reducing the amount of vegetation in the forest canopy and understory (commonly referred to as “clutter”) which can obstruct fly-ways and interfere with echolocation. Previous studies have shown that several species of insectivorous bats will avoid foraging in clutter, [27], [28], [29], as this can reduce foraging success [30]. Second, disturbance from fire increases abundance of insect prey. Post-fire growth of early successional plant species increases terrestrial insect activity [25], [31], [32], [33] and shifts the community composition and increasing abundance of emergent aquatic insects in streams [34], [35]. These changes in insect populations are likely to benefit bat foraging, though recent work suggests that the structural characteristics of forest habitat following controlled burning may have primacy over prey availability for many forest bat species [36]. Third, fire is assumed to increase the quantity and quality of roosting habitat by creating dead and dying trees, and perhaps by facilitating disease and decay in live trees as well. Tree roosting occurs in late successional features, such as under exfoliating bark and in crevices in dead and live trees [37], [38], [39], [40], [41]; and prescribed fire has been shown to increase roosting opportunities relative to adjacent unburned forest [24]. The relative importance of fire extent and severity on each of these three responses is largely unstudied.

To date, a single study has examined the effect of wildfire on bat activity using recorded bat echolocation calls [35]. Greater bat activity was observed in high-severity burned riparian habitat within mixed-confer forest than at unburned areas of similar habitat in central Idaho. However, species and foraging guilds were not differentiated in this study, relegating inference to overall bat activity. Due to the large range of variation in wing morphology, echolocation frequency, foraging behavior, and roosting habitat requirements among forest bat species, it is likely responses to habitat change vary among species, suggesting that a multispecies approach to assessing the impacts of wildfire on forest bats is prudent and important in identifying management-relevant responses.

Forest bat species are likely adapted to use spatially complex mosaics of forest patches, with early successional stages being important to foraging and late successional stages being necessary for roosting [42]. This suggests that a mosaic of burn severity, and subsequent succession, on the landscape could be important for the maintenance of diverse bat communities. Most investigations on the relationship between ecological disturbance and bat activity have been at the stand-level, but recent findings from landscape scale studies suggest that the importance of early successional patches may vary with scale [42], [43].

We conducted a study on a ∼61,000 ha wildfire area in the southern Sierra Nevada mountain range of California, USA. The wildfire burned with mixed-severity, leaving a mosaic of fire damage [44], [45]. The forests of this region are known to support communities of bats comprised of up to 16 species [46]. Our objective was to evaluate the effects of fire severity on bat activity in mixed-conifer forest. We compared relative activity of six phonic groups (including individual species and approximate feeding guilds) of bats across unburned and burned stands experiencing moderate and high tree canopy mortality (i.e. moderate- to high-severity) one year post-fire. To examine stand vs. landscape scale effects of fire, we also analyzed bat activity in unburned stands with varying levels of high-severity fire in the surrounding area.

## Methods

### Study Area

**Figure 1:**
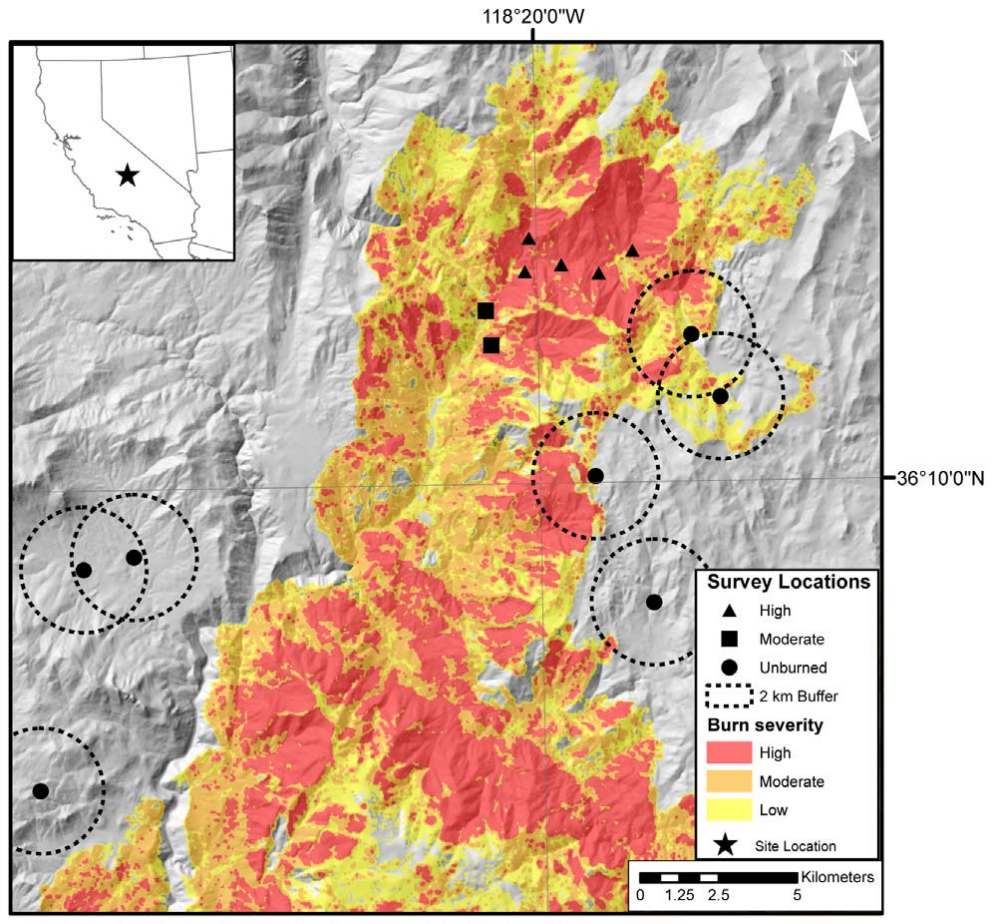
Study area. Map of sampled portion of the 2002 McNally Fire (southern Sierra Nevada mountains, California, USA) with topography and the extent of low-, moderate-, and high-severity fire damaged areas with study sites identified by severity type. Dashed lines represent 2 km radii buffers used to quantify the area of high-severity fire damage surrounding each unburned site.

We conducted our study in the Sequoia and Inyo National Forests in the southern Sierra Nevada of California, USA (Latitude: 36°10′37″N, Longitude: 118°20′13″W; Fig. 1). The study area is mountainous, with elevations ranging from 1,570 m to 2,575 m. The vegetation community is characterized as Sierran, mixed-conifer forest; consisting of mixed or individually dominated stands of red and white fir (*Abies magnifica, A. concolor*); Jeffrey, ponderosa, and sugar pine (*Pinus jeffreyi, P. ponderosa, P. lambertiana*, respectively); and incense cedar (*Calocedrus decurrens*) [47]. Canopies range from closed to open, often with shrubs in the understory.

The McNally Fire burned approximately 60,985 ha in the Sequoia and Inyo National Forests, including 33,704 ha of conifer-dominated forests from 22 July –27 August 2002 following an anthropogenic ignition under initially severe weather conditions [45]. The McNally fire was of mixed-severity, leaving a mosaic of low to high-severity damage, including patches of unburned forest [44], [45].

### Sampling of Bat Activity

We compared echolocation activity of bats in three levels of disturbance: unburned, moderate- (40–89% canopy scorch), and high-severity (>90% canopy scorch; see below for detail) burn in riparian and upland forests to assess how activity differed in relation to burn-status and habitat type (Fig. 1). Burned survey locations were chosen randomly prior to sampling using ArcGIS (Environmental Research Systems Institute, Redlands, CA) random point generator and then finding the closest suitable site (e.g. a site with no vegetative or acoustic interference) from the randomly generated coordinates. Locations in burned habitat were constrained to occur within the McNally fire perimeter, in mixed-conifer habitats based on existing data layers of California vegetation type [48], and within a single watershed (9 Mile Creek, 2,005 ha) due to the logistical challenges of sampling mountainous terrain. Unburned areas adjacent to the fire perimeter were assumed to represent pre-burn conditions and were chosen randomly from the same mixed-conifer habitat type. Upland and riparian locations were paired to test for differences in how fire may have affected bats that have preferential use for riparian versus upland habitat types. We selected 14 sites total, each with an upland and riparian pair, seven within the burn area and seven in unburned habitats. Burned sites included two stands which burned at moderate severity and five stands which burned at high severity (Fig. 1, Table S1).

Surveys were conducted from July through August, 2003, one year after the fire; this corresponds to a timeframe when female bats are reproductive and resource requirements highest [49]. We recorded ultrasonic echolocation calls of bats using Anabat II detectors connected to compact flash ZCAIM data storage units (Titley Electronics, Ballina, Australia). Bat activity was recorded all night from sunset to sunrise at each site for 2 to 9 (mean = 6) nights (Table S1). Each survey site consisted of one detector placed at a stream edge, perpendicular to the stream corridor to sample activity within the riparian corridor and a paired detector located 75–100 m away in adjacent upland habitat. Half of the sites were surveyed during a 9 day period in late July and the second half during a 6 day period in early August.

We sampled unburned and burned areas simultaneously to minimize variation in echolocation activity, as bat activity is known to vary temporally due to prey availability and weather conditions [50], [51]. To reduce bias in detection probability, we standardized our survey methods between the two disturbance regimes. Detectors were placed in forest gaps, mounted 1 m above the ground, and oriented 45 degrees off horizontal to reduce signal attenuation by understory vegetation. Detectors were calibrated to equal sensitivity [52]. Required permits for field surveys conducted within Sequoia and Inyo National Forests were obtained from the U.S. Forest Service (USFS). Because acoustic survey is a completely non-invasive sampling technique, this project required no institutional approval regarding animal care or use.

Analook (C. Corben; http://www.hoarybat.com) was used to visually classify bat calls to species and phonic groups. We could not categorize all calls to species due to similarities in calls among species with similar call morphology. We partitioned calls into one of six phonic groups based on call characteristics (e.g., pulse duration and terminal frequency of the call sweep [53]). Three groups consisted of single species which are reliably identified by the uniqueness of their call morphology, including pallid bat (*Antrozous pallidus*, ANPA), fringed myotis (*Myotis thysanodes,* MYTH), and long-eared myotis (*Myotis evotis,* MYEV). The remaining three phonic groups consisted of species which are not reliably identified by the uniqueness of their call morphology and were categorized based on the terminal frequency of the call sweep, including LB25, MY50, and MY40. Bats in the LB25 group are “large-bodied” species with narrowband echolocation calls terminating at approximately 25 KHz, assumed to represent Mexican free-tailed bats, (*Tadarida brasiliensis*), big brown bats (*Eptesicus fuscus*), hoary bats (*Lasiurus cinereus*), and silver-haired bats (*Lasionycteris noctivagans*). Bats in the MY50 (50 KHz range) group represent Yuma myotis (*Myotis yumanensis*) and California myotis (*Myotis californicus*). Bats in the MY40 (40 KHz range) group represent little brown bat (*Myotis lucifugus*), long-legged myotis (*Myotis volans*), and small-footed myotis (*Myotis ciliolabrum*). Call classifications were made by a single person to minimize observer bias.

Bat activity was quantified as the number of passes per night at each survey location [54]. A pass was defined as a series of echolocation calls separated by more than 1.5 seconds. Because feeding buzzes were not recorded frequently, we used the combined number of feeding buzzes and search phase calls to estimate activity. We sampled bat activity on 162 detector-nights and recorded 11,097 bat passes which could reliably be assigned to a phonic group.

### Measurement of Fire Severity and Landscape Covariates

Fire damage was classified as unburned (0–10% canopy change), low severity (fire-caused crown scorch affected <40% overstory canopy foliage), moderate-severity (crown scorch of 40–89% of forest canopy) or high-severity (crown scorch or loss of 90% of canopy) based on USFS vegetation burn-severity maps [55] and Burn Area Emergency Rehabilitation (BAER) classifications using Landsat 7 and SPOT multi-spectral satellite imagery immediately pre- and post-fire [45], [56]. No survey sites occurred in low severity burn areas, thus our analysis was limited to assessing the effects of moderate- to high-severity fire on bat activity one year following fire.

### Statistical Analyses

Acoustic detectors do not detect all bat species equally, leading to under-representation of some species in surveys [57]. We limited analysis to comparisons within phonic groups across levels of disturbance, making no attempt to compare the relative activity levels among phonic groups. Inference is restricted to comparing differences within phonic groups. Activity data showed signs of zero inflation and overdispersion and we examined a range of analytical methods, including zero inflated Poisson and negative binomial regression, as well as ordinal transformations of the data to estimate the effects of habitat and fire severity on bat activity. Data were best represented by pooling across nights for each site and calculating the mean number of passes per night at each site followed by a natural log transformation to conform to assumptions of normality for an analysis of variance (supplemental figure S1). While this approach reduced statistical power by collapsing data into 28 sample units instead of 162 units per phonic group (the number of nights sampled), it permitted a simpler and more easily interpreted two-way analysis of variance (ANOVA) of habitat and fire severity on bat activity and was qualitatively the same. We found no evidence of violation of homogeneity of variance based on inspection of residuals. We found little support for interactions of habitat and fire severity, based on Akaike information criteria (AIC) and *F*-tests of maximum likelihood ratios, and thus only present results of additive models.

To assess how fire at the landscape scale affects bat activity, we analyzed unburned sites to identify differences relative to the extent of high-severity fire damage within a 2 km radius. This scale was selected based on previous studies detecting landscape-scale effects of forest disturbance on other vertebrates [58]. We used ArcGIS to calculate the percentage of high-severity burned habitat within a 2 km radius surrounding each unburned survey location according to USFS vegetation burn-severity maps (Fig. 1) [55]. We used simple linear regression on unburned sites with a predictor of the proportion of the area within 2 km burned with high-severity. Parameter estimates and their 95% confidence intervals are presented to demonstrate effects of fire severity and habitat on each phonic group. Parameter estimates reflect the effect of fire severity relative to unburned conditions or upland relative to riparian habitat. All analyses were conducted in R 2.13 [59].

## Results

**Figure 2:**
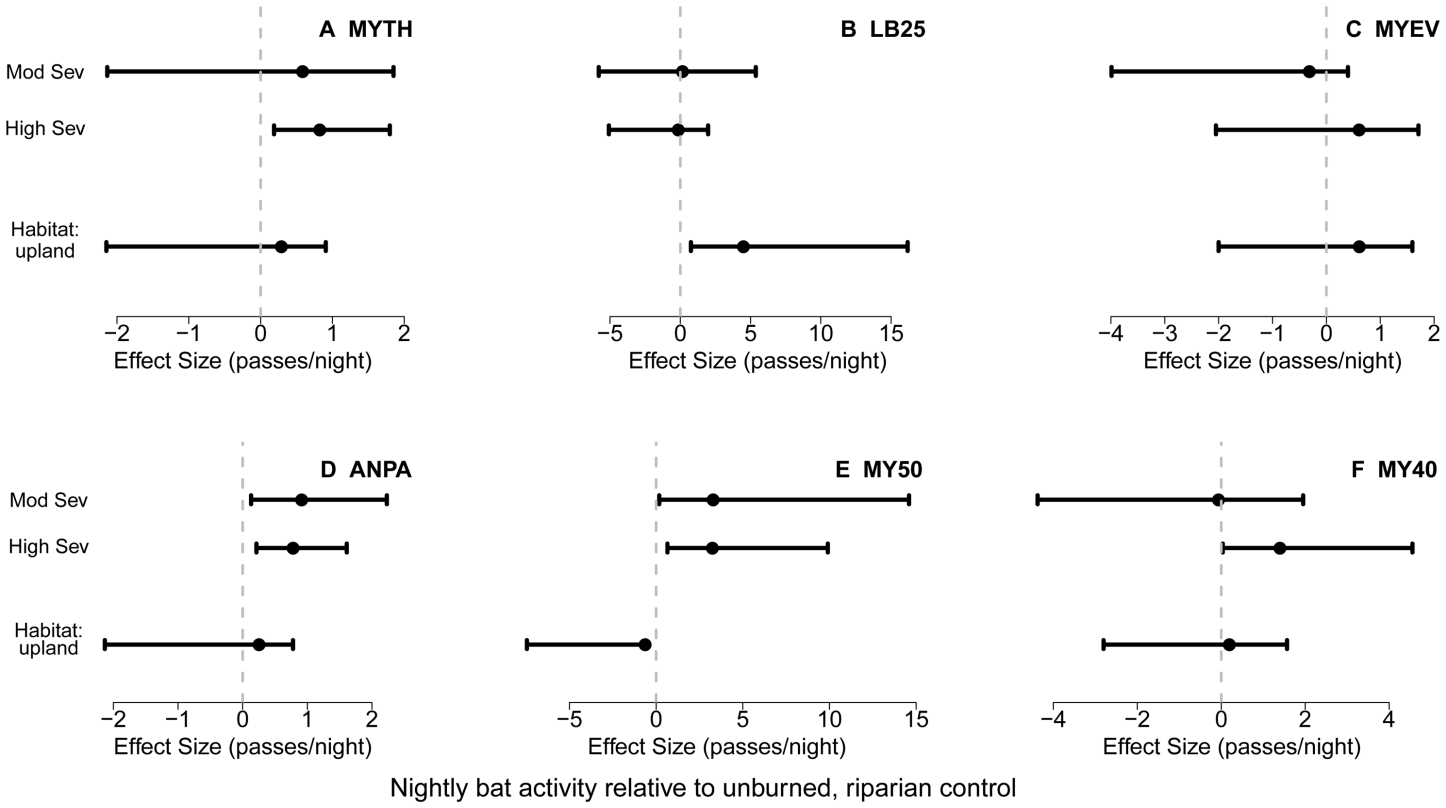
Effect of fire severity and habitat on nightly bat activity. Effect size (natural log-transformed number of calls per night and 95% confidence intervals) of moderate- and high-severity fire, as well as the effect of habitat (upland vs. riparian), on bat activity one year post-fire in mixed-conifer forest of California. Effects are relative to unburned, riparian forest stands among (A) *Myotis thysanodes* = MYTH; (B) “large-bodied” species in the 25 KHz range = LB25; (C) *Myotis evotis* = MYEV; (D) *Antrozous pallidus* = ANPA; (E) *Myotis* species in the 50 KHz range = MY50; and (F) *Myotis* species in the 40 KHz range = MY40.

Bat activity in burned areas was either equivalent or higher than in unburned stands of mixed-conifer forest for all six phonic groups (Fig. 2, Table S1). Of the six phonic groups, four groups had activity levels significantly greater in burned stands than in unburned stands in at least one level of fire severity (Fig. 2, Table S2). Two phonic groups (*M. thysanodes*, MY40) showed differing response to fire severity with positive response to high-severity fire and neutral response to moderate-severity (Fig. 2A, F). Four of the six phonic groups showed no differences in activity among riparian and upland habitat types (Fig. 2). Activity in the phonic group MY50, which includes *M. yumanensis*, a riparian specialist, was higher in riparian habitat. In contrast, activity in LB25 phonic group, composed of large-bodied bat species, was higher in upland habitats (Fig. 2B, E). Effect size estimates for fire and habitat were of a similar magnitude, ranging from differences of ∼ 0 to 5 passes per night relative to unburned stands. Activity in unburned stands varied widely with phonic group, generally ranging from a few passes per night for *M. evotis* or *A. pallidus* to more than 180 for LB25 (Table S1).

**Figure 3:**
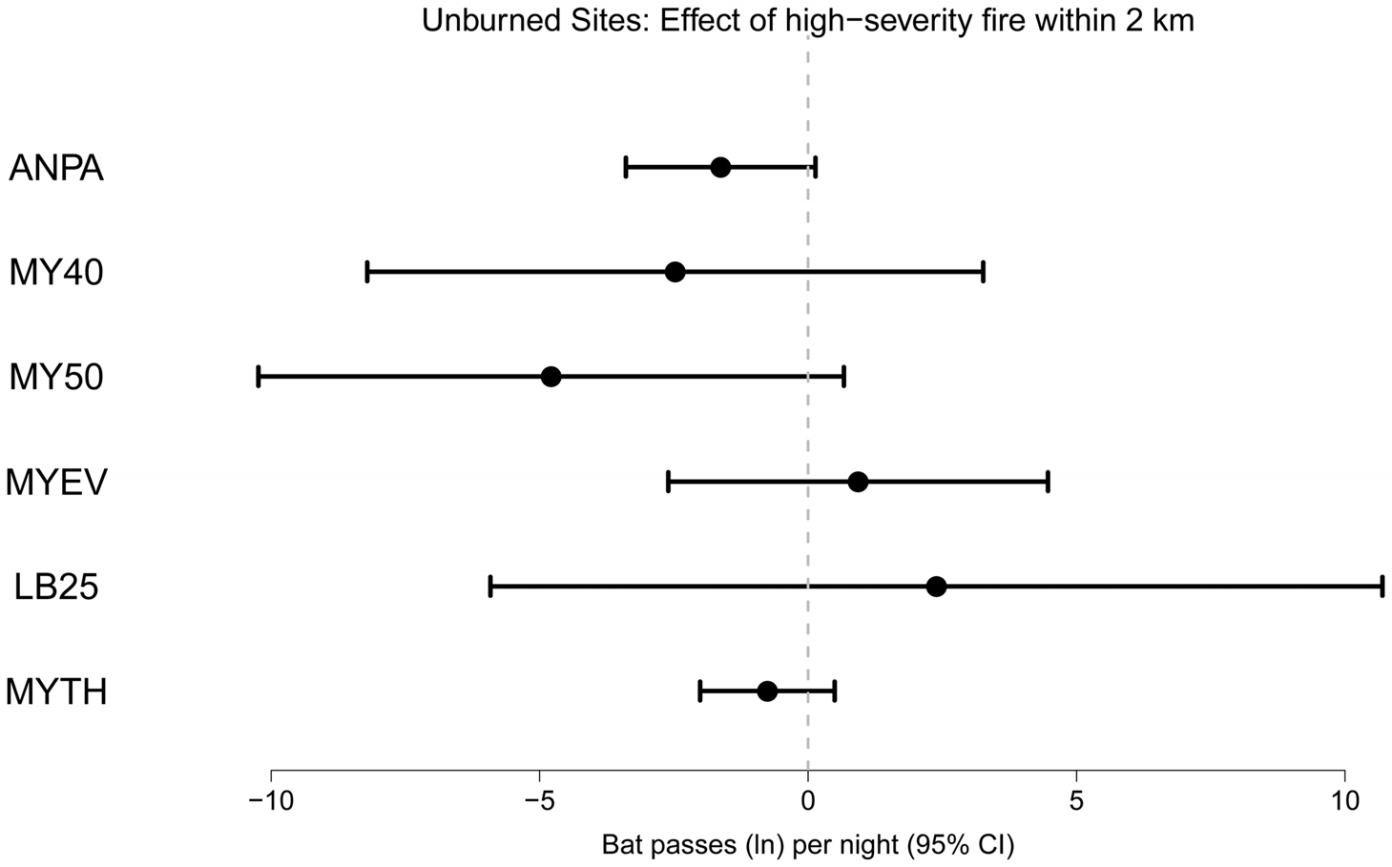
Effect of the landscape fire mosaic on nightly bat activity. Effect size (natural log-transformed number of calls per night and 95% confidence intervals) of the amount of stand-replacing fire within a 2 km radius on bat activity in unburned forest stands.

We found no statistical differences in activity among unburned stands differing in their landscape fire context (Fig. 3, Table S3) for any phonic group. The extent of high-severity fire within 2 km of unburned sites ranged from 0 to 30% of the surrounding landscape (Fig. 1).

## Discussion

The importance and widespread nature of mixed-severity fire across a broad range of forest types in western North America is increasingly being recognized [11]. However, the response of many species to fire and the role of fire in provisioning habitat are poorly studied [9]. This work documents the response of different bat species groupings to wildfire along a fire severity and habitat gradient in mixed-conifer forest. To our knowledge, this is the first study to document taxa-specific and severity-specific response of bats, a vertebrate group of high conservation significance [60]. Bat response was categorically neutral to positive one year after wildfire suggesting that bats are resilient to wildfire and that naturally generated early successional habitats are an important landscape component for bats as has been demonstrated for birds [8], [9] and a range of plant taxa [5], [61]. Our results suggest response of bats to wildfire in the southern Sierra Nevada in California varies among species, but that most phonic groups show higher activity in areas burned with moderate- to high-severity. Increased abundance and unique community composition [8] or persistence of species thought to be sensitive to fire [1] have been documented for birds in post-fire landscapes. Thus, the effects of mixed-severity burns appear to be particularly important for vagile wildlife, including bats, which are well suited to exploit a mosaic of forest patches at differing stages of succession [42].

The positive response of most phonic groups to recently burned landscapes broadly mirrors findings for a range of bird species adapted to foraging and nesting in burned forest habitat substrates. Avian response to fire severity has been classified into a range of response patterns across species, including flat, linear (increasing, decreasing) and peaked (e.g. Fig. 2 in [62]). Our data suggest a similar conceptual framework is relevant for bats. Two phonic groups (*M. thysanodes* and MY40) demonstrated increasing magnitude of response with severity, two groups (*A. pallidus* and MY50 showed a positive threshold response to fire (no differentiation of fire severity but positive fire response), and two groups (LB25 and *M. evotis*) showed no response (Fig. 2). We encourage use of this framework in future studies as a basis to predict response patterns and to investigate underlying causal mechanisms.

Mounting evidence suggests that fire-prone forests and associated fauna are often resilient to stand-replacing fire [1], [2], [9], [63]. In this study, we found no significant negative effects of fire on bat activity in a mixed-conifer forest one year after a large and severe wildfire, supporting the view that forest bat communities are resilient to fire and that fire may enhance foraging opportunities. Although phonic groups within this study demonstrated clear heterogeneity in habitat use, patterns of use did not differ across fire severity. This result suggests that the factors that drive use of forest habitats (e.g. foraging opportunity, prey species) were functionally equivalent post-fire, reaffirming the resilience of the system and bat phonic groups to landscape-scale mixed-severity fire in fire-prone forests of Western North America.

The wildfire-landscape mosaic did not affect bat activity in unburned stands for any of the six phonic groups. Some stands had up to 30% of the surrounding landscape within a 2 km radius burned with stand replacement fire. Despite this, activity was neither higher (due to immigration of species preferring unburned conditions) nor lower (due to emigration to favored habitat conditions elsewhere), suggesting that bat communities do not respond to forest landscape condition in a manner similar to that documented for territorial birds following fire [64]. Rather, bats are likely foraging and roosting across much broader spatial scales [65], resulting in greater resilience to changes at this scale. However, caution is warranted as this study did not explicitly examine roosting habitat or patterns of daily foraging commuting as could be done with telemetry [41].

Insect response to fire has been studied in a range of settings with well-documented neutral to positive impacts on a several taxa of flying insects [25], [66], including Pyrophilous species (i.e. those favored by fire) spanning 25 families from 4 insect orders (Hemiptera, Diptera, Coleoptera, Lepidoptera). Pyrophilous species tend to be most abundant in the first 1–3 years following fire as they exploit fire-killed wood, fungi, and heightened availability of nutrients following fire [67]. Indeed, dynamics of fire-adapted positive responding wood herbivores are especially well-studied [66]. Many of these insect taxa represent potential prey for bat communities occupying conifer forests in western North America [68]. Aquatic insects have also been observed to increase in abundance post-fire. Malison and Baxter [34] observed greater insect emergence in riparian habitat that experienced high-severity wildfire versus low-severity and unburned sites. While tracking insect emergence, Malison and Baxter also observed the greatest number of bat echolocation calls at high-severity sites, arguing that wildfire may lead to an extended ‘‘fire pulse’’ stimulating aquatic productivity (taxa such as Chironomidae, *Baetis* spp., and Simuliidae) [35], a pattern of bat activity consistent with the results of our study. The phonic group MY50, which includes the riparian specialist species *M. yumanensis* with documented prey preferences for emergent insects [53], [69], showed the greatest activity levels in riparian habitat and in habitats burned at moderate- to high-severity (Fig. 2). While further research is required, evidence from prescribed fires in eastern North America suggests the importance of post-fire prey availability on bat foraging activity [25], [36], and we hypothesize that increased abundance of flying insects played an important role in the patterns observed in this study.

The positive response of bats to fire broadly mirrors a range of bird species adapted to foraging in open conditions and not dependent on live conifer foliage for foraging or nesting substrates. Despite the broad parallels found in this study with avian post-fire response, further work exploring a broader range of post-fire conditions is necessary such as time since fire and influence of pre-fire forest condition in multiple forest types. This research reflects the first documented taxa-specific response of bats to a single wildfire. Further work to investigate response of bats on other wildfires and in other fire-sensitive ecosystems at varying stages of succession is needed to broaden the scope of inference from our results, as other research investigating avian and reptile responses to fire at multiple spatial scales in non-forested habitats of Australia have found late-successional conditions favored by birds [70] and mid-successional stages favored by reptiles [13]. Furthermore, focused work investigating prey availability, changes in foraging efficiency, and the effects of fire on roosting behavior are also warranted. Broadly, the link between acoustic detectability, habitat type and use, and actual bat density is a topic needing further research.

### Conclusions

Our results support the emerging perspective that naturally generated early successional habitats are essential on the landscape for a broad range of taxa and that processes like wildfire are instrumental in their maintenance. Our results, in conjunction with the only other peer-reviewed study on bat response to wildfire [35] and numerous studies on prescribed fire, strongly suggest that occurrence of fire on the landscape is an important process for maintenance of forest bat communities, as it appears to be for many other vertebrate species [9], [71] and forest processes [72]. Fire-generated early successional conditions can harbor unique assemblages of species not found elsewhere [8] and in some regions represent the rarest habitat types on the landscape (e.g. Pacific Northwest Forests) [61]. Similar to recent findings which suggest the importance of retaining unlogged conditions in logged landscapes for maintenance of bat foraging and roosting habitat [19], restoring fire as a process to fire-prone forests may be equally important to the proper management of forest bat communities. This growing body of evidence should guide forest management with regard to restoration activities such as prescribed fire and “let-burn” policies, as well as post-fire management. This study represents a first step in providing land managers with the necessary information to anticipate the effects of large wildfires on forest bat communities and to incorporate these expectations into fire management plans on publicly owned lands.

## Supporting Information

Figure S1 **Distribution of bat activity by phonic group in relation to burn severity.** Natural log-transformed boxplot and dot plots of each phonic group by level of disturbance (i.e., high- and moderate-severity wildfire and unburned) among (A) *Myotis thysanodes* = MYTH; (B) “large-bodied” species in the 25 KHz range = LB25; (C) *Myotis evotis* = MYEV; (D) *Antrozous pallidus* = ANPA; (E) *Myotis* species in the 50 KHz range = MY50; and (F) *Myotis* species in the 40 KHz range = MY40.
(TIF)

Table S1 **Acoustic survey information summarized for 14 survey locations with paired detectors deployed at each location in different habitats designated by habitat type (upland and riparian).**
(DOCX)

Table S2 **Modeling results for effects of fire severity and habitat on bat phonic group activity one year after wildfire in mixed-conifer forest, California, USA.**
(DOCX)

Table S3 **Modeling results for the effect of landscape-scale fire on bat activity in unburned forest in mixed-conifer forest one year post-fire, California, USA.**
(DOCX)
