# Supplementary figures and images for: Bat Response to Differing Fire Severity in Mixed-Conifer Forest California, USA
Source: PLoS One. 2013 Mar 6;8(3):e57884. doi: 10.1371/journal.pone.0057884 (PMC3590284; doi:10.1371/journal.pone.0057884)

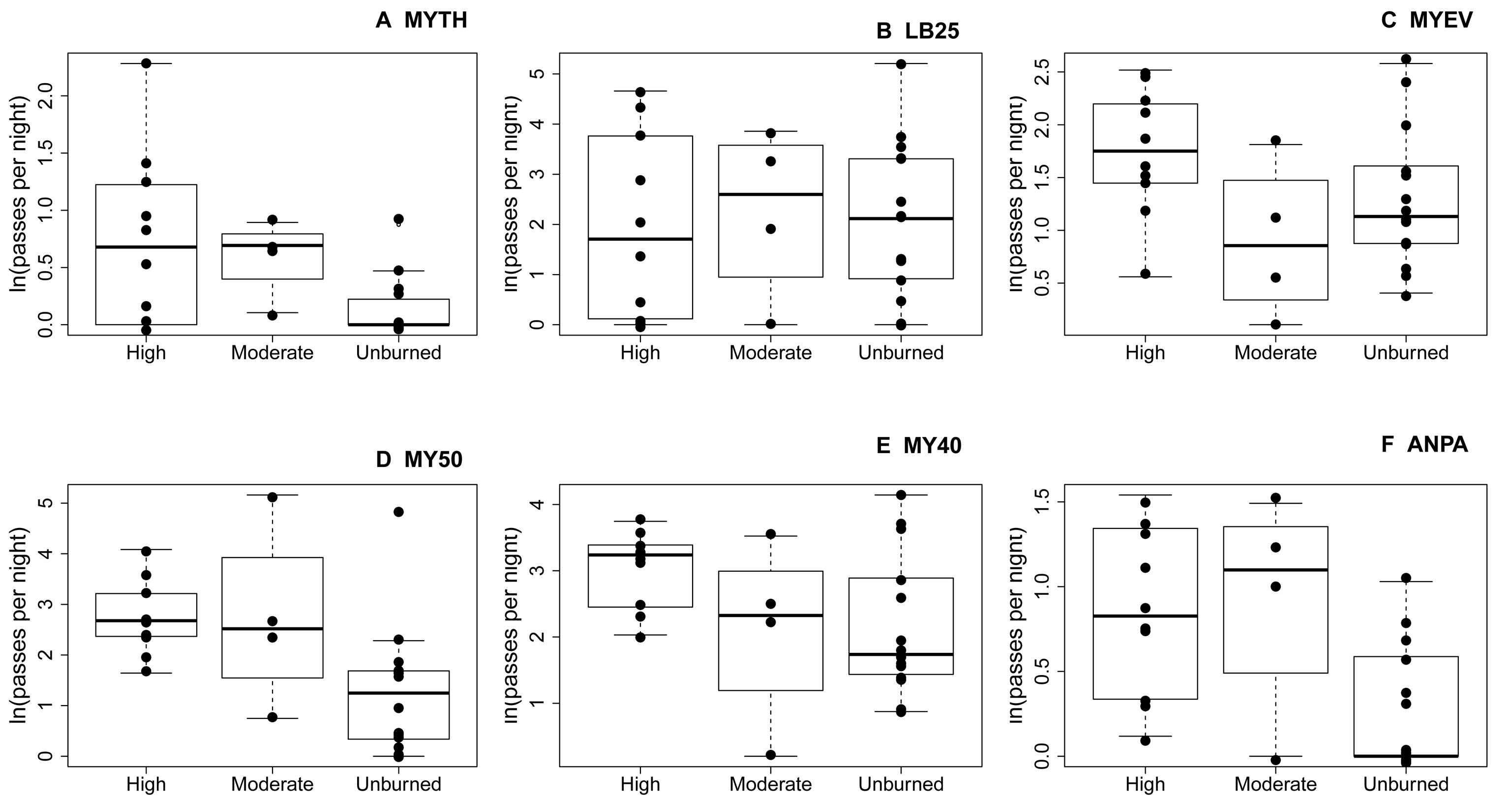

Supplement: Figure S1 — Distribution of bat activity by phonic group in relation to burn severity. Natural log-transformed boxplot and dot plots of each phonic group by level of disturbance (i.e., high- and moderate-severity wildfire and unburned) among (A) Myotis thysanodes = MYTH; (B) “large-bodied” species in the 25 KHz range = LB25; (C) Myotis evotis = MYEV; (D) Antrozous pallidus = ANPA; (E) Myotis species in the 50 KHz range = MY50; and (F) Myotis species in the 40 KHz range = MY40. (TIF) [file pone.0057884.s001.tif]
