# Supplementary material for: Bat Response to Differing Fire Severity in Mixed-Conifer Forest California, USA
Source: PLoS One. 2013 Mar 6;8(3):e57884. doi: 10.1371/journal.pone.0057884 (PMC3590284; doi:10.1371/journal.pone.0057884)
Supplement: Table S1 — Acoustic survey information summarized for 14 survey locations with paired detectors deployed at each location in different habitats designated by habitat type (upland and riparian). (DOCX) [file pone.0057884.s002.docx]

| Survey location | Habitat type | Severity | Elevation (m) | No. of nights surveyed | Mean no. of calls recorded per night per phonic group* | | | | | |
| --- | --- | --- | --- | --- | --- | --- | --- | --- | --- | --- |
|  |  |  |  |  | MYTH | MYEV | MY40 | MY50 | LB25 | ANPA |
| 1 | Upland | Unburned | 2,380 | 5 | 0 | 3.6 | 41.4 | 0.6 | 2.6 | 0 |
|  | Riparian |  |  | 5 | 0 | 2.2 | 12.8 | 4.4 | 0.6 | 0 |
| 2 | Upland | Unburned | 1,703 | 5 | 0 | 0.8 | 3.0 | 0.2 | 1.6 | 1.0 |
|  | Riparian |  |  | 5 | 0.3 | 1.4 | 1.4 | 4.0 | 24.2 | 1.8 |
| 3 | Upland | Unburned | 1,710 | 5 | 1.4 | 4.0 | 3.2 | 1.6 | 4.6 | 1.2 |
|  | Riparian |  |  | 5 | 0 | 1.4 | 1.6 | 5.6 | 32.8 | 0.4 |
| 4 | Upland | Unburned | 2,048 | 5 | 0.6 | 2.6 | 5.2 | 0.6 | 16.0 | 0.8 |
|  | Riparian |  |  | 2 | 0 | 0.5 | 62.0 | 124.0 | 1.0 | 0.5 |
| 5 | Upland | Unburned | 2,348 | 5 | 0 | 12.2 | 6 | 0 | 6.6 | 0 |
|  | Riparian |  |  | 3 | 0 | 9.7 | 17.0 | 3.7 | 26.0 | 0 |
| 6 | Upland | Unburned | 2,542 | 5 | 0 | 0.8 | 4.2 | 0.4 | 180.2 | 0 |
|  | Riparian |  |  | 5 | 0 | 2 | 1.4 | 0 | 0 | 0 |
| 7 | Upland | Unburned | 2,575 | 5 | 0.4 | 6.0 | 35.2 | 8.8 | 1.2 | 0 |
|  | Riparian |  |  | 5 | 0 | 2.0 | 3.6 | 4.4 | 0 | 0 |
|  |  | Mean | 2,187 | 5 | 0.2 | 3.5 | 14.1 | 11.3 | 21.2 | 0.4 |
|  |  |  |  |  |  |  |  |  |  |  |
| 8 | Upland | Moderate | 1,570 | 8 | 1.0 | 5.1 | 10.8 | 13.8 | 33.1 | 2.4 |
|  | Riparian |  |  | 9 | 1.0 | 0.8 | 32.9 | 173.3 | 1.0 | 1.7 |
| 9 | Upland | Moderate | 1,610 | 9 | 1.4 | 2.1 | 7.9 | 9.4 | 16.0 | 3.4 |
|  | Riparian |  |  | 9 | 0.1 | 0.1 | 0.2 | 1.1 | 0 | 0 |
| 10 | Upland | High | 1,887 | 8 | 0 | 3.3 | 28.6 | 13.8 | 4.5 | 1.4 |
|  | Riparian |  |  | 8 | 0 | 0.8 | 34.1 | 23.9 | 0.1 | 0.1 |
| 11 | Upland | High | 2,017 | 5 | 8.8 | 11.4 | 21.4 | 5.8 | 1.4 | 0.4 |
|  | Riparian |  |  | 5 | 2.4 | 5.8 | 25.8 | 14.0 | 0 | 0.4 |
| 12 | Upland | High | 1,771 | 8 | 0.1 | 3.9 | 6.6 | 13.4 | 36.9 | 1.0 |
|  | Riparian |  |  | 5 | 0 | 2.4 | 9.0 | 35.0 | 0 | 2.0 |
| 13 | Upland | High | 1,918 | 6 | 3.2 | 7.5 | 41.3 | 4.2 | 58.8 | 3.0 |
|  | Riparian |  |  | 6 | 1.3 | 10.5 | 25.0 | 9.7 | 12.8 | 2.8 |
| 14 | Upland | High | 1,855 | 6 | 0.7 | 8.0 | 24.0 | 10.0 | 82.2 | 3.7 |
|  | Riparian |  |  | 6 | 1.6 | 3.8 | 10.6 | 58.4 | 0 | 1.2 |
|  |  | Mean | 1,804 | 7 | 1.5 | 4.7 | 20.6 | 25.2 | 19.0 | 1.7 |

*MYTH = *Myotis thysanodes*; MYEV = *Myotis evotis*; MY40 = *Myotis* species in the 40 KHz range; MY50 = *Myotis* species in the 50 KHz range; LB25 = “large-bodied” species in the 25 KHz range; ANPA = *Antrozous pallidus*.
