# Supplementary material for: Bat Response to Differing Fire Severity in Mixed-Conifer Forest California, USA
Source: PLoS One. 2013 Mar 6;8(3):e57884. doi: 10.1371/journal.pone.0057884 (PMC3590284; doi:10.1371/journal.pone.0057884)
Supplement: Table S2 — Modeling results for effects of fire severity and habitat on bat phonic group activity one year after wildfire in mixed-conifer forest, California, USA. (DOCX) [file pone.0057884.s003.docx]

| Phonic Group | Factor | Parameter Estimate† | SE | t-value | P-value |
| --- | --- | --- | --- | --- | --- |
| MYTH | Moderate | 0.46 | 0.28 | 1.6 | 0.12 |
|  | High | 0.60 | 0.21 | 2.9 | 0.01 |
|  | Habitat | 0.25 | 0.19 | 1.3 | 0.19 |
| LB25 | Moderate | 0.14 | 0.83 | 0.2 | 0.87 |
|  | High | -0.16 | 0.60 | 0.3 | 0.8 |
|  | Habitat | 1.70 | 0.55 | 3.1 | 0.005 |
| MYEV | Moderate | -0.38 | 0.35 | 1.1 | 0.28 |
|  | High | 0.47 | 0.25 | 1.9 | 0.07 |
|  | Habitat | 0.48 | 0.23 | 2.1 | 0.05 |
| MY50 | Moderate | 1.46 | 0.62 | 2.3 | 0.03 |
|  | High | 1.45 | 0.46 | 3.2 | 0.004 |
|  | Habitat | -1.00 | 0.42 | 2.4 | 0.02 |
| MY40 | Moderate | -0.07 | 0.56 | 0.1 | 0.91 |
|  | High | 0.88 | 0.41 | 2.2 | 0.04 |
|  | Habitat | 0.18 | 0.37 | 0.5 | 0.64 |
| ANPA | Moderate | 0.65 | 0.25 | 2.6 | 0.02 |
|  | High | 0.58 | 0.19 | 3.1 | 0.005 |
|  | Habitat | 0.23 | 0.17 | 1.3 | 0.19 |

†Units of parameter estimate are ln-transformed passes/night.
