# Supplementary material for: Bat Response to Differing Fire Severity in Mixed-Conifer Forest California, USA
Source: PLoS One. 2013 Mar 6;8(3):e57884. doi: 10.1371/journal.pone.0057884 (PMC3590284; doi:10.1371/journal.pone.0057884)
Supplement: Table S3 — Modeling results for the effect of landscape-scale fire on bat activity in unburned forest in mixed-conifer forest one year post-fire, California, USA. (DOCX) [file pone.0057884.s004.docx]

| Phonic Group | Parameter Estimate† | SE | t-value | P-value |
| --- | --- | --- | --- | --- |
| MYTH | -0.76 | 0.59 | -1.3 | 0.22 |
| LB25 | 2.39 | 3.88 | 0.6 | 0.55 |
| MYEV | 0.93 | 1.65 | 0.6 | 0.58 |
| MY50 | -4.79 | 2.55 | -1.9 | 0.09 |
| MY40 | -2.47 | 2.68 | -0.9 | 0.38 |
| ANPA | -1.63 | 0.83 | -2.0 | 0.07 |

†Units of parameter estimate are ln-transformed passes/night.
